# Циклический синдром Кушинга: трудности диагностического поиска и выбора тактики лечения. Клинический случай

**DOI:** 10.14341/probl13140

**Published:** 2023-02-25

**Authors:** Л. К. Дзеранова, А. В. Доровских, Е. А. Пигарова, А. М. Лапшина, С. Ю. Воротникова, А. С. Шутова, М. А. Перепелова, А. Ю. Григорьев, В. Н. Азизян

**Affiliations:** Национальный медицинский исследовательский центр эндокринологии; Национальный медицинский исследовательский центр эндокринологии; Национальный медицинский исследовательский центр эндокринологии; Национальный медицинский исследовательский центр эндокринологии; Национальный медицинский исследовательский центр эндокринологии; Национальный медицинский исследовательский центр эндокринологии; Национальный медицинский исследовательский центр эндокринологии; Национальный медицинский исследовательский центр эндокринологии; Национальный медицинский исследовательский центр эндокринологии

**Keywords:** циклический синдром Кушинга, болезнь Иценко-Кушинга, эндогенный гиперкортицизм, АКТГ-эктопированный синдром

## Abstract

Циклический синдром Кушинга — патологическое состояние, для которого характерно чередование периодов ­избыточной секреции кортизола с соответствующими клиническими проявлениями и периодов спонтанной ремиссии заболевания.

Считается, что для установления диагноза эндогенного гиперкортицизма циклического течения необходимо зафиксировать не менее трех эпизодов чрезмерной секреции кортизола, сменяющихся периодами нормализации его продукции.

В большинстве случаев указанную патологию диагностируют у пациентов с АКТГ-секретирующей опухолью гипофиза, однако в литературе описаны редкие случаи циклической формы гиперкортицизма при наличии эктопированной секреции АКТГ опухолями разной локализации, а также без явной верификации очага патологической гормональной секреции. Кроме того, циклическая гиперпродукция кортизола может наблюдаться и при АКТГ-независимом синдроме Кушинга, ассоциированном с наличием кортикостеромы или гиперплазии надпочечников. Точные причины и механизмы циклического течения гиперкортицизма в настоящее время изучены недостаточно.

В связи с нетипичным течением заболевания, непредсказуемостью возникновения нового «цикла», вариабельностью его длительности и проявлений (не только у разных пациентов, но и у одного и того же пациента) верификация диагноза и определение тактики лечения могут быть затруднены в повседневной практике специалистов, а распространенность указанного состояния приуменьшена.

## Abstract (en)

Cyclic Cushing’s syndrome is a pathological condition characterized by alternating periods of excessive cortisol secretion with corresponding clinical manifestations and periods of spontaneous remission of the disease.

To diagnose Cyclic Cushing’s syndrome it is necessary to record at least three episodes of excessive cortisol secretion alternating with periods of normalization of its production.

In most cases, this pathology is diagnosed in patients with ACTH-secreting pituitary tumor, however, there are rare cases of cyclic hypercorticism with ectopic ACTH secretion by tumors of different localization and without verification of pathological hormonal secretion focus. In addition, cyclic hyperproduction of cortisol can be also observed in ACTH-independent Cushing’s syndrome associated with the presence of corticosteroma or adrenal hyperplasia. The exact causes and mechanisms of the cyclic hypercorticism are currently insufficiently studied.

Due to the atypical course of the disease, the unpredictability of the occurrence of a new «cycle», the variability of its duration and manifestations (not only in different patients, but also in the same patient), verification of the diagnosis and determination of treatment tactics may be difficult in the daily practice of specialists, and the prevalence of this condition can be undervalued.

## АКТУАЛЬНОСТЬ

Первое описание циклической формы гиперкортицизма было опубликовано в конце 50-х годов прошлого века [1], однако интерес к синдрому Кушинга перемежающего течения не исчезает и в настоящие время.

Среди возможных причин данного состояния выделяют дисфункцию гипоталамуса, патологическую реакцию кортикотропных клеток на нейротрансмиттеры (такие как кортиколиберин, дофамин, нейроэпинефрин, серотонин, γ-аминомасляную кислоту), спонтанное кровоизлияние в опухоль гипофиза с последующим нарушением синтеза гормонов кортикотрофами, патологию регуляторных механизмов отрицательной обратной связи в системе гипоталамус-гипофиз-надпочечники, некоторые другие. Точные механизмы возникновения периодов, сопровождающихся гиперкортицизмом, сменяющихся нормализацией гормональной секреции, еще предстоит выяснить [2].

По разным данным, циклический синдром Кушинга встречается в 15–36% случаев эндогенного гиперкортицизма [3]. С учетом вариабельности частоты и продолжительности фаз заболевания (от нескольких часов до нескольких месяцев), непредсказуемости возникновения нового «цикла» гиперкортицизма и спонтанной нормализации гормонального статуса, разнообразия клинических проявлений и степени их выраженности, циклический синдром Кушинга — одна из самых серьезных и актуальных проблем современной эндокринологии.

В данной статье мы сообщаем о случае циклической формы болезни Кушинга, обсуждаем трудности диагностического поиска, выбора тактики лечения, а также полученные результаты.

## ОПИСАНИЕ КЛИНИЧЕСКОГО СЛУЧАЯ

Пациентка С., 57 лет.

С ноября 2017 г. стала отмечать изменения внешности в виде отечности лица и прибавки массы тела без изменения привычной диеты. Исходно и на фоне прибавки массы тела индекс массы тела (ИМТ) — норма, нарушений углеводного обмена и артериальной гипертензии (АГ) в анамнезе не было. Обращало внимание распределение жировых отложений преимущественно в области лица и живота. Кроме того, пациентку беспокоили жалобы неврологического характера (онемение конечностей, нарушения координации движений), с которыми обратилась к неврологу. По результатам проведенной МРТ головного мозга в 2018 г. выявлена микроаденома гипофиза размерами 9×6×6 мм (рис. 1). Гормональные исследования на предмет активности новообразования не проводили, специфического лечения назначено не было.

В течение полугода состояние пациентки ухудшилось: появились судороги нижних конечностей, выраженная слабость и утомляемость, эпизоды повышения артериального давления (АД) до 160/100 мм рт. ст. В мае 2018 г. по результатам лабораторного обследования зафиксированы повышение уровня адренокортикотропного гормона (АКТГ) утром до 145 пг/мл (при норме до 46,0 пг/мл), отсутствие подавления кортизола в ходе малой дексаметазоновой пробы (кортизол сыворотки крови 548,8 нм/л). По данным большой дексаметазоновой пробы — снижение кортизола более чем на 50%. С учетом полученных данных и выявленной ранее аденомы гипофиза, установлен диагноз эндогенного гиперкортицизма центрального генеза. Дополнительно проведена КТ органов брюшной полости (заключение: без патологии). В сентябре того же года в областной больнице по месту жительства выполнена трансназальная аденомэктомия (гистологическое, иммуногистохимическое (ИГХ) исследования не проводились). В послеоперационном периоде пациентка отмечала мышечную и общую слабость, инициирована терапия гидрокортизоном в дозе 5 мг в сутки. При контрольном обследовании через 1 мес после операции АКТГ утром в сыворотке крови 50,0 пг/мл (норма до 46,0 пг/мл), через 2 мес — 31,0 пг/мл, кортизол утром — 146 нмоль/л. Через 3 мес после оперативного лечения с учетом отсутствия клинико-лабораторных признаков надпочечниковой недостаточности терапия глюкокортикостероидами (ГКС) отменена, пациентка отмечала улучшение общего состояния.

В июне 2020 г., на фоне сильного эмоционального стресса, повторно возникли жалобы на изменение внешности по типу «лунообразного лица», значительное повышение аппетита, увеличение массы тела на 8 кг за 6 мес без изменения диеты, выраженную слабость. По данным лабораторных исследований — рецидив гиперкортицизма: кортизол слюны, собранной в 23.00, — 69 (0,5–9,65) нмоль/л, кортизол в суточной моче — 2433,0 (100–379) нмоль/сут, АКТГ в сыворотке крови утром 166 (2–25,5) пг/мл, кортизол — 712 (64–327) нмоль/л. По данным МРТ головного мозга от июля 2020 г. (рис. 2) послеоперационные изменения селлярной области, без динамики по сравнению с 2018 г. Специфического лечения назначено не было, была направлена на консультацию в ФГБУ «НМИЦ эндокринологии» Минздрава России, где в сентябре 2020 г. при обследовании наблюдался регресс лабораторных признаков гиперкортицизма: выявлено незначительное повышение кортизола слюны, собранной в 23.00, — 10,28 ммоль/л (норма до 9, 65 нмоль/л), кортизол в суточной моче — в пределах референсных значений, результат малой дексаметазоновой пробы положительный — кортизол 45,0 нмоль/л. Данных за наличие нарушения углеводного обмена получено не было (глюкоза сыворотки крови — 4,68 ммоль/л). По результату рентгеновской денситометрии — без существенных изменений. Также наблюдались исчезновение внешних проявлений эндогенного гиперкортицизма и отсутствие характерных для данного заболевания жалоб. Несмотря на отсутствие полной лабораторной ремиссии, в связи с компенсацией клинической картины повторное хирургическое лечение показано не было, рекомендовано динамическое наблюдение.

В июле 2021 г. перенесла новую коронавирусную инфекцию (COVID-2019) средней степени тяжести. C августа того же года вновь стала отмечать ухудшение самочувствия в виде повышения аппетита, прибавки массы тела (в течение 3 мес на 7 кг), нарушения сна, общей слабости, онемения конечностей, болей в суставах. По данным лабораторных исследований: декомпенсация по основному заболеванию (кортизол слюны в 23.00 — 27,0 нмоль/л, АКТГ утром — 90 пг/мл, кортизол на фоне малой дексаметазоновой пробы — 596 нмоль/л). При повторной госпитализации в ФГБУ «НМИЦ эндокринологии» Минздрава России в ноябре 2021 г.: кортизол в слюне вечером — 13,46 (0,5–9,65) нмоль/л, кортизол в суточной моче — 398 (100–379) нмоль/сут, ритм АКТГ (утро/вечер) — 63,55/36,65 пг/мл. Проведено МРТ-исследование головного мозга с контрастным усилением (рис. 3), выявлена макроаденома гипофиза размерами 6×9×11 мм с эндо-, латероселлярным распространением вправо, по шкале Knosp-IV (полное окружение кавернозного сегмента внутренней сонной артерии опухолью). При оценке минеральной плотности кости (МПК) методом рентгеновской денситометрии верифицированы остеопения в поясничном отделе позвоночника (-1,3 по Т-критерию) и в левой бедренной кости (-1,0 по Т-критерию), остеопороз дистальной трети левой лучевой кости (-3,1 по Т-критерию). Проведен консилиум совместно с заведующим отделением нейрохирургии, учитывая особенности размеров и характера роста опухоли, отсутствие полной лабораторной ремиссии при наличии ремиссии клинической, было принято решение о динамическом наблюдении и проведении трансназальной транссфеноидальной аденомэктомии на фоне выраженной активности основного заболевания.

С января 2022 г. снова стала отмечать ухудшение состояния, когда появились признаки гиперкортицизма. При амбулаторном обследовании: кортизол в суточной моче — 2664,0 нмоль/сут, АКТГ — 123 пг/мл, кортизол в сыворотке крови — 940 нмоль/л, кортизол в слюне в 23.00 — 29,9 нмоль/л. В марте 2022 г. в ходе госпитализации в отделение нейроэндокринологии ФГБУ «НМИЦ эндокринологии» Минздрава России лабораторно подтвержден рецидив эндогенного гиперкортицизма: кортизол свободный в моче — 2557,6 нмоль/сут (100–379), кортизол слюны в 23.00 — 17,52 нмоль/л (0,5–9,65), кортизол крови вечером — 704,4 нмоль/л (64–327), АКТГ утром — 102,8 пг/мл (7,2–63,3), АКТГ вечером — 71,7 пг/мл (2–25,5). По данным МРТ головного мозга (рис. 4): эндо-латероселлярная аденома гипофиза с распространением вправо по шкале Knosp-IV, размерами 8×12×10 мм (умеренное увеличение размеров в сравнении с исследованием от 20.11.2021 г.). 29 марта 2022 г. выполнено повторное оперативное лечение в объеме трансназального транссфеноидального удаления аденомы гипофиза. По результату ИГХ-исследования удаленной ткани: плотногранулированная кортикотропинома (оценка экспрессии Ki-67 в данном случае не проводилась). В послеоперационном периоде состояние пациентки удовлетворительное, выраженных жалоб не предъявляла.

**Figure:**
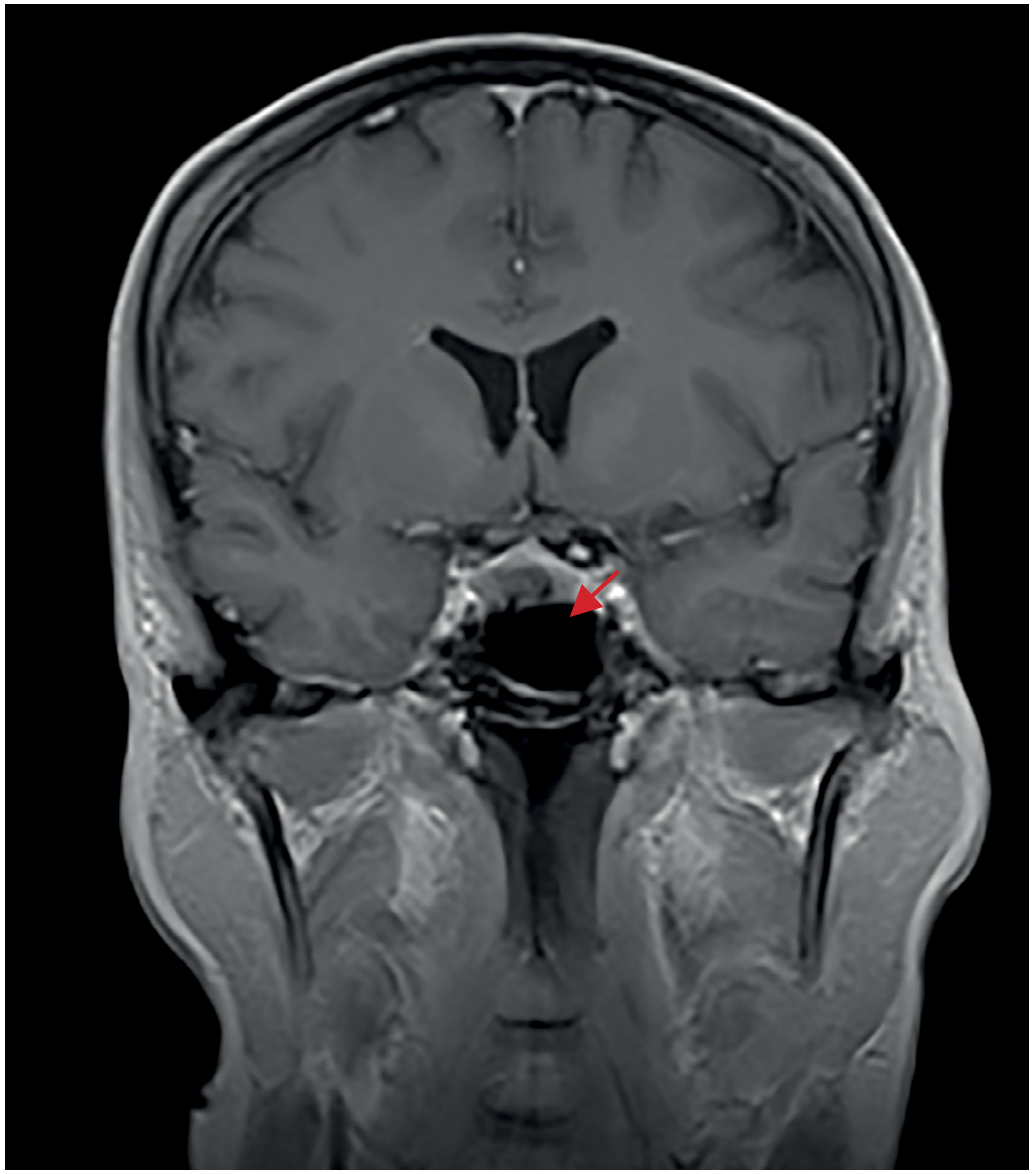
Рисунок 1. МРТ головного мозга до операции (трансназальной аденомэктомии), 2018 г.

**Figure:**
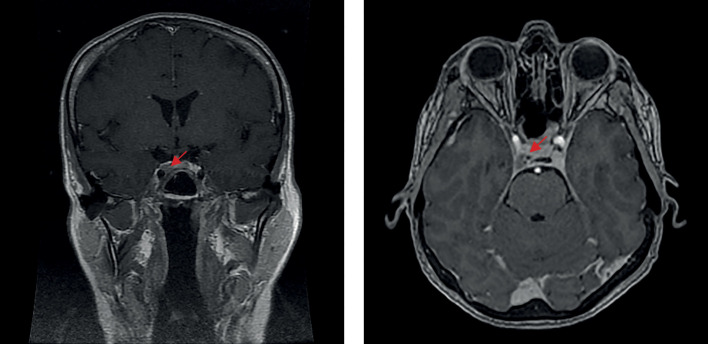
Рисунок 2. МРТ головного мозга после трансназальной аденомэктомии, 2020 г.

**Figure:**
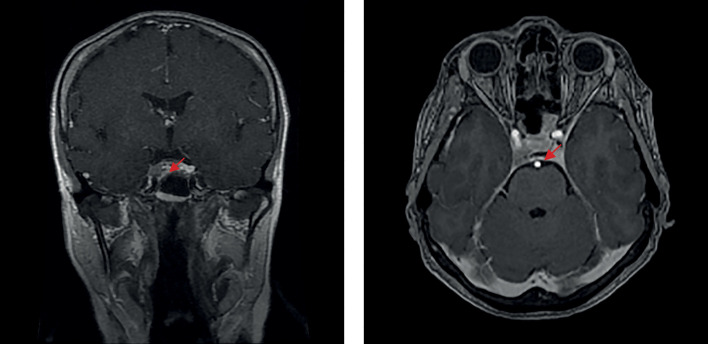
Рисунок 3. МРТ головного мозга после трансназальной аденомэктомии, 2021 г.

**Figure:**
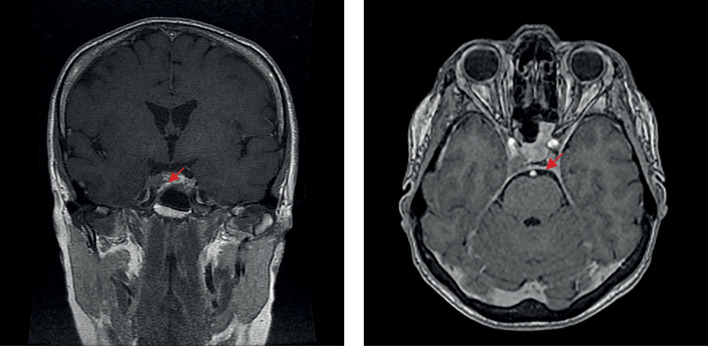
Рисунок 4. МРТ головного мозга перед второй операцией (повторной трансназальной аденомэктомией), 2022 г.

## ОБСУЖДЕНИЕ

В большинстве случаев клиническая картина заболевания, обусловленного чрезмерным действием эндогенного кортизола, специфична и постоянна. Основные проявления гиперкортицизма длительного течения представлены характерным перераспределением жировой ткани, повышением артериального давления, системным остеопорозом, дисфункцией половой системы, иммунодефицитным состоянием, нарушением трофики кожных покровов, миопатией, нарушением углеводного обмена [3]. В приведенном клиническом случае с учетом циклического течения заболевания, выражающегося чередованием эпизодов гиперсекреции кортизола и периодов нормализации гормонального профиля, клинические проявления были временны и довольно неспецифичны, что вызывало сомнения при установке диагноза.

Считается, что необходимо зафиксировать не менее трех пиков и двух спадов выработки кортизола для верификации синдрома Кушинга циклического течения [2]. Принимая во внимание четыре зафиксированных при проведении лабораторных исследований «цикла» гиперкортицизма, диагноз эндогенного гиперкортицизма интермиттирующего течения не вызывает сомнения.

Следует учитывать, что, как и при классической форме эндогенного гиперкортицизма, циклическая гиперкортизолемия может быть вызвана гормональной активностью кортикотропиномы (в 80–85% случаев), редко — эктопическим высвобождением кортикотропина или независимыми от секреции АКТГ причинами (при наличии аденомы или карциномы коры надпочечников, двусторонней макро- или микроузловой гиперплазии) [5].

При верификации диагноза АКТГ-зависимого гиперкортицизма на основании результатов лабораторных исследований для определения дальнейшей тактики лечения необходимо выявить источник патологической секреции АКТГ. АКТГ-секретирующая аденома гипофиза, как правило, подчиняется принципу обратной отрицательной связи, что демонстрируется снижением уровня кортизола более чем на 50% исходного при применении больших доз ГКС (в ходе проведения большой дексаметазоновой пробы), АКТГ-эктопированная ткань не обладает указанным свойством. Однако чувствительность данного метода диагностики достаточно низкая, так как при наличии макроаденомы гипофиза и при полной автономии патологической ткани секреция АКТГ аденомой гипофиза может не подавляться большими дозами ГКС [6]. Кроме того, в ряде случаев секреция АКТГ эктопированными образованиями подавляется большими дозами ГКС по принципу обратной отрицательной связи [7].

По данным литературы, для АКТГ-эктопированного синдрома возможно более тяжелое течение заболевания, но данное проявление неспецифично. Высокое значение АКТГ в вечернее время (более 110 пг/мл) наблюдается чаще при эктопии очага секреции АКТГ (в 70%) [8], тем не менее данный лабораторный показатель также неоднозначный.

С целью топической диагностики рекомендовано проведение МРТ головного мозга, и в большинстве случаев подтверждается наличие аденомы гипофиза. При размерах аденомы гипофиза более 6 мм у пациентов с эндогенным гиперкортицизмом может быть установлен диагноз болезни Иценко–Кушинга, в то время как наличие новообразования менее 6 мм в диаметре не всегда свидетельствует об источнике избыточной секреции АКТГ. Согласно данным литературы, инциденталома гипофиза указанных размеров обнаружена у 10–20% здоровых людей [9][10]. По результатам исследований частота инциденталом гипофиза составляет 14,4% по данным аутопсии и 22,5% при МРТ-скрининге [11]. В данном случае результатами МРТ-исследования первично была подтверждена аденома гипофиза размерами более 6 мм, что позволило установить диагноз болезни Иценко–Кушинга и выбрать хирургическое лечение как приоритетный вариант. Косвенными дополнительными признаками правильности выбора послужили также положительная большая дексаметазоновая проба и отсутствие нарушения ритма секреции АКТГ.

При отсутствии патологии гипоталамо-гипофизарной области требуется проведение дополнительного инструментального обследования. Эктопированный очаг циклической секреции АКТГ может быть различной локализации: в легких, тимусе, поджелудочной железе, щитовидной железе, надпочечнике, яичнике, почке, желудке, аппендиксе, обладать как доброкачественным, так и злокачественным потенциалом [12, 13]. Известны также редкие случаи АКТГ-секреции параганглиомой, нейробластомой, хемодектомой [14]. Следовательно, при подозрении на АКТГ-эктопированный синдром идентификация очага патологической секреции АКТГ может быть затруднительной и еще более сложной — при циклическом течении заболевания.

Среди методов инструментальной диагностики применяют КТ органов грудной клетки, средостения, брюшной полости, малого таза [15]. В случае получения отрицательных результатов КТ рекомендовано рассмотреть возможность выполнения сканирования организма с помощью сцинтиграфии рецепторов соматостатина в режиме Total Body с ¹¹¹In-октреотидом, 123I-метайодбензилгуанидином или 99mTc-тектротидом и/или совмещенную позитронно-эмиссионную (ПЭТ) и компьютерную томографию (КТ) с DOTA конъюгированным радиофармпрепаратом (68Ga-DOTA-TATE) [4].

В настоящее время наиболее информативный метод дифференциальной диагностики АКТГ-секретирующей аденомы гипофиза и АКТГ-эктопированного синдрома представлен двусторонним одномоментным селективным забором крови из нижних каменистых синусов (чувствительность метода 88–100%) [16][17]. В приведенном примере показаний к проведению указанного инвазивного исследования выявлено не было, проведение повторной трансназальной аденомэктомии обусловлено наличием рецидива основного заболевания, подтвержденного данными инструментального обследования (при МРТ головного мозга: макроаденома гипофиза), отсутствием динамики роста образования легкого по данным динамического наблюдения.

Таким образом, выбор метода лечения циклического синдрома Кушинга зависит от локализации очага патологической секреции. Хирургическое вмешательство считается наиболее эффективным и предпочтительным методом лечения при установленном наличии кортикотропиномы. Успех оперативного лечения зависит от многих факторов: размеров и особенностей клеточного строения опухоли, степени прорастания в кавернозные синусы, а также от квалификации нейрохирурга. Трансназальная аденомэктомия, по разным данным, позволяет достичь ремиссии в 65–90% случаев. Однако возникновение рецидива заболевания не исключено.

Показателем ремиссии основного заболевания служит развитие надпочечниковой недостаточности. В случае циклического течения гиперкортицизма сложность заключается в том, что данные лабораторных показателей в послеоперационном периоде могут быть схожими с показателями, соответствующими завершению «цикла» гиперкортицизма, что требует дальнейшего длительного наблюдения.

При сохранении активности заболевания после проведенного хирургического лечения или невозможности проведения полной резекции опухолевой ткани гипофиза необходимо рассмотреть несколько вариантов дальнейшего лечения: повторное оперативное вмешательство, лучевая терапия и/или непрерывная фармакотерапия. Решение должно обсуждаться многопрофильной медицинской бригадой в индивидуальном порядке.

Так, лучевая терапия представляет собой эффективный метод лечения в случаях отсутствия возможности полного иссечения аденомы гипофиза, а также при рецидивах болезни Кушинга. Наиболее распространенными методами являются стереотаксическая радиохирургия и фракционированная внешняя лучевая терапия. Следует учитывать, что наступление эффекта от данного метода лечения может быть отсрочено (на несколько месяцев/лет), а риск возникновения гипопитуитаризма значителен [5].

Среди медикаментозной терапии в настоящее время в зарубежных исследованиях описана эффективность многих фармакологических препаратов: ингибиторов стероидогенеза (метирапона, кетоконазола, митотана, этомидата, осилодростата), препаратов, воздействующих на глюкокортикоидные рецепторы (мифепристона), и препаратов, оказывающих влияние на гипофизарную ткань (каберголина, пасиреотида) [5]. Важно указать, что в Российской Федерации зарегистрированные показания для осуществления медикаментозного контроля эндогенного гиперкортицизма среди указанных препаратов имеет только пасиреотид. Возможность использования остальных препаратов, несмотря на патофизиологическую обоснованность, должна обсуждаться с пациентами индивидуально [4].

В случаях эктопированного очага секреции АКТГ необходимо оценить возможность проведения его хирургической резекции. При невозможности выполнения оперативного лечения (например, в условиях отсутствия визуализации очага патологической секреции АКТГ, при метастатическом распространении) стоит рассмотреть вариант медикаментозной терапии (аналогами соматостатина, блокаторами стероидогенеза) [5][18].

Если состояние особенно тяжелое и/или вышеуказанные методы не оказались достаточно эффективными, а также в условиях неверифицированной локализации очага секреции АКТГ двусторонняя адреналэктомия остается потенциально спасающим жизнь вариантом лечения [2].

Поскольку признаки и симптомы эндогенного гиперкортицизма как классического, так и циклического течения имеют тенденцию прогрессировать с течением времени, и заболевание в конечном итоге может привести к летальному исходу, лечение указанного патологического состояния не следует откладывать.

## ЗАКЛЮЧЕНИЕ

Циклический синдром Кушинга — редкая, трудно диагностируемая форма эндогенного гиперкортицизма ввиду непостоянности клинических проявлений и противоречивости лабораторных. Возникновение и продолжительность нового эпизода гиперкортицизма непредсказуемы, межцикловые периоды могут существенно варьироваться по длительности (от нескольких дней до месяцев). По этиологии заболевание может быть как центрального (в большинстве случаев), так и периферического генеза, что еще больше затрудняет верификацию диагноза и выбор стратегии дальнейшего лечения.

В приведенном клиническом случае представлены данные о четырех зафиксированных эпизодах гиперкортицизма, сменяющихся периодами нормализации гормонального профиля, что позволяет сделать вывод о циклической форме болезни Кушинга. С учетом верификации аденомы гипофиза размерами более 6 мм по результатам МРТ, проведение оперативного лечения в объеме трансназальной аденомэктомии обосновано. Развитие надпочечниковой недостаточности в послеоперационном периоде свидетельствовало об успешности проведенного хирургического лечения. Однако, принимая во внимание возникновение рецидива заболевания, подтвержденного клиническими и лабораторно-диагностическими данными, требовалось продолжение лечения. Выбор в пользу повторного оперативного вмешательства на фоне регресса заболевания обоснован наличием макроаденомы гипофиза по данным МРТ головного мозга, а также возможностью проведения операции в высокоспециализированном стационаре. Результаты лабораторного обследования в послеоперационном периоде (развитие надпочечниковой недостаточности, регресс клинических проявлений, улучшение общего самочувствия) свидетельствуют в пользу эффективности проведенного лечения. Данными ИГХ-исследования удаленной ткани подтверждено наличие кортикотропиномы. В настоящее время продолжается амбулаторное наблюдение пациентки.

Таким образом, циклический синдром Кушинга — диагностическая дилемма, с которой врачи могут столкнуться в клинической практике. В данном случае требуются особо тщательное динамическое наблюдение пациента и оценка клинико-лабораторных данных. Описание клинических случаев гиперкортицизма циклического течения имеет важное значение для формирования представления о специфике заболевания, возможностях его диагностики и лечения.

С ростом числа пациентов, имеющих избыточную массу тела или ожирение, клиницистам следует быть еще более внимательными при подозрении на наличие гиперкортицизма по данным анамнеза, особенно при получении противоречивых результатов обследования. В сомнительных случаях требуется проводить динамическое лабораторно-инструментальное обследование с целью исключения патологии, фиксирование субъективных симптомов гиперкортицизма, чтобы не пропустить редкую, но тяжелую патологию.

## ДОПОЛНИТЕЛЬНАЯ ИНФОРМАЦИЯ

Источники финансирования. Работа выполнена по инициативе авторов без привлечения финансирования.

Конфликт интересов. Авторы декларируют отсутствие явных и потенциальных конфликтов интересов, связанных с содержанием настоящей статьи. Все авторы одобрили финальную версию статьи перед публикацией, выразили согласие нести ответственность за все аспекты работы, подразумевающую надлежащее изучение и решение вопросов, связанных с точностью или добросовестностью любой части работы.

Согласие пациента. Авторы настоящей статьи получили письменное разрешение от упоминаемых в статье пациентов на публикацию их медицинских данных в журнале «Проблемы эндокринологии».
